# Supplementary material for: Exploring the association between housing insecurity and mental health among renters: A systematic review of quantitative primary and secondary studies
Source: PLoS One. 2026 Jan 16;21(1):e0294222. doi: 10.1371/journal.pone.0294222 (PMC12810827; doi:10.1371/journal.pone.0294222)
Supplement: Attachment — Submitted filename: Response to Reviewers.docx [file pone.0294222.s008.docx]

PONE-D-23-35202

Exploring the association between housing insecurity and mental health among renters: A systematic review

**Response to Reviewers**

**Academic Editor:** After careful consideration, we feel that it has merit but does not fully meet PLOS ONE’s publication criteria as it currently stands. Therefore, we invite you to submit a revised version of the manuscript that addresses the points raised during the review process. The reviewers have provided helpful information regarding an assortment of things concerning your manuscript.  A consistent concern among both reviewers was the need to expand your lit review sources.  Still, it appears that both reviewers feel the manuscript deserves some consideration once these edits are made.

**Reply:** *Thank you for your guidance. We have carefully considered all suggestions and incorporated additional relevant literature to strengthen the manuscript. We are encouraged by the reviewers' acknowledgment of the manuscript's potential and refined it to meet PLOS ONE's publication criteria.* *We believe that these revisions have significantly improved the manuscript and hope it is now suitable for publication.*

**Reviewer #1:** This article is very interesting. I make some comments on it:

**Reply:** *Thank you for your insightful comments on our manuscript. We greatly appreciate your feedback and have made efforts to incorporate your suggestions diligently. Below, we outline the changes we have implemented in response to your comments.*

Main comments

**1.** Introduction and discussion: To understand why access to housing is a problem nowadays it is important to present a conceptual framework of the causes, eg: public housing policies and the market. Many countries have left the access to housing to the market and therefore prices have increased and the accessibility has decreased. Moreover public spending on housing is very low. The paper should mention that. See for example:
-Marí-Dell'Olmo M, Novoa AM, Camprubí L, Peralta A, Vásquez-Vera H, Bosch J, Amat J, Díaz F, Palència L, Mehdipanah R, Rodríguez-Sanz M, Malmusi D, Borrell C. Housing Policies and Health Inequalities. Int J Health Serv. 2017 Apr;47(2):207-232. doi: 10.1177/0020731416684292. Epub 2016 Dec 28. PMID: 28030990.

**Reply:** *Thank you for emphasizing the importance of presenting a conceptual framework to address the causes of housing access challenges. In response to your feedback, we have revised both the introduction and discussion (practical implications) to spotlight the primary macrostructural factors influencing housing insecurity and to offer an overview of the current state of housing accessibility in OECD countries.*

**Changes in manuscript (p. 3f.):** “Housing insecurity is profoundly influenced by various macrostructural factors, including the welfare state, housing system, public housing policies, and market dynamics (Marí-Dell'Olmo et al., 2017). In their paper, Marí-Dell’Olmo et al. (2017) provide a conceptual framework on housing systems, housing conditions, and health equity, illustrating how these structural dimensions interact and impact access to adequate housing, as well as subsequent physical and mental health outcomes. Despite the diverse housing systems, regulatory environments, and socio-economic factors among countries, many advanced economies share a common trajectory of severe housing insecurity challenges (Lee et al., 2022). This review focuses on OECD countries for several reasons: They typically exhibit similar economic structures characterized by market-based economies with varying levels of government intervention, and they often adopt comparable housing policies and regulations, albeit with variations in their scope and implementation, such as social housing programs and tenant protections. Furthermore, in many OECD countries, heavy reliance on the market for access to housing has led to rising prices and reduced accessibility of housing, exacerbating the challenges for renters in securing affordable, stable, and suitable housing (Baumgartner et al., 2023; Bone, 2014; Clair et al., 2019; Eurostat, 2022; Routhier, 2019) (…) Moreover, public expenditure on housing remains low across many OECD countries, resulting in a heavy reliance on the often minimally regulated private rental market for access to affordable housing (Del Salvi Peroi et al., 2016). Although some governments have implemented a range of housing policies and programs to address the issue of housing security, including social/public housing, housing subsidies, or eviction policies, these initiatives frequently fail to reach those who truly need them, leaving many eligible renters without the necessary support (DeLuca & Rosen, 2022; Dweik & Woodhall-Melnik, 2022) (…) within OECD countries.“

**Changes in manuscript (p. 34f.): “**At the same time, our findings emphasize the critical need to enhance access to affordable and stable housing options for renters, especially low-income households, as a means to combat housing insecurity and enhance public mental health. Structural factors such as welfare state policies, housing systems, and market dynamics significantly influence housing insecurity and insufficient public expenditure on housing exacerbates this issue (Del Salvi Peroi et al., 2016; Lee et al., 2022; Marí-Dell'Olmo et al., 2017). Therefore, prioritizing policy responses to address housing insecurity is vital for promoting the well-being and resilience of affected individuals and communities. However, identifying effective housing policy measures, particularly for vulnerable renter groups such as low-income, disabled, or racially marginalized households, requires further research and investigation (DeLuca & Rosen, 2022).”

**2.** Introduction, results and discussion: Housing insecurity change by the axes of inequality (gender, social class, race…). It is necessary to deeper emphasize this reality. Do the papers found analyse this fact? See for example:
-Vásquez-Vera C, Fernández A, Borrell C. Gender-based inequalities in the effects of housing on health: A critical review. SSM Popul Health. 2022 Mar 11;17:101068. doi: 10.1016/j.ssmph.2022.101068. PMID: 35360438; PMCID: PMC8961216.

**Reply:** *Thank you for your highlighting the fact that inequalities such as gender, social class, and race intersect with housing insecurity and its impact on health outcomes. We have underscored this intersection in our introduction and provided a more detailed discussion outline: Regrettably, the studies included in our analysis mostly did not specifically address or explore this aspect but rather examined the entire group of renters collectively. We acknowledge this as a significant gap in the current literature and have emphasized it in our text to suggest avenues for future research in response to your commentary in more detail (both in the introduction and discussion).*

**Changes in manuscript (p. 4): “**(…) as well as dimensions of inequality and discrimination such as social class, race, disability, and gender, creating a complex interplay in which these interact and mutually reinforce each other (Hulse & Saugeres, 2008; C. Vásquez-Vera et al., 2022).”

**Changes in manuscript (p. 33): “**(…) such as gender, social class, disability, and race (Bentley et al., 2019; Carrere et al., 2022; DeLuca & Rosen, 2022; Hulse & Saugeres, 2008; Kirkpatrick & Tarasuk, 2011; Morris et al., 2017; C. Vásquez-Vera, Fernández, & Borrell, 2022). (…) For instance, in a review examining gender-based inequalities in the effects of housing on health, findings suggest that while negative impacts are observed across all genders regarding housing affordability, instability, and insecurity, women, non-binary, and transgender people tend to experience more pronounced effects (C. Vásquez-Vera, Fernández, & Borrell, 2022). However, the studies included in our analysis exclusively examined renters as a whole. Recognizing the heterogeneity within this group and the likelihood of varied experiences of housing insecurity and its impact on mental health based on economic and social circumstances, it is essential for future research to adopt an intersectional lens and a bidirectional approach (cf. conceptual framework proposed by C. Vásquez-Vera, Fernández, and Borrell, 2022). Studies further indicate that housing insecurity and its impact on mental health also varies on a macro-level, across different social, legal, and cultural contexts (Acolin, 2022; Bentley et al., 2016; Herbers & Mulder, 2017; Hulse & Milligan, 2014; Hulse et al., 2011; C. Vásquez-Vera, Fernández, & Borrell, 2022) (…).”

**3.** Results: I am not sure that tables 1 and 2 have to be inside the paper, they can be in an appendix. In the results section I would include a table showing the descriptive results found: the number of papers for each variable (including the mental health results).

**Reply:** *We agree that the results section benefits from a descriptive table, as tables 1 and 2 are quite long and may make it challenging for the reader to quickly grasp the descriptive results. Therefore, we have followed your proposal and added a table showing the descriptive results. Regarding the placement of tables 1 and 2 (now tables 2 and 3), we defer to the editor's decision. However, personally, we would opt to leave these tables inside the paper, as advised in the PRISMA guidelines, as they provide essential information for our paper.*

**Changes in manuscript (p. 11):**

**Table 1**

*Descriptive of studies included*

|  | **N°**  **(*N* = 22)** | **%** |
| --- | --- | --- |
| **Year of Publication**  2009  2010 – 2019  2020 – 2023 | 1  8  13 | *4,5*  *36,4*  *59,1* |
| **Country**  US  Australia  UK  South Korea  Spain  The Netherlands | 11  6  3  1  1  1 | *50,0*  *27,3*  *13,6*  *4,5*  *4,5*  *4,5* |
| **Study design**  Longitudinal  Cross-sectional  Quasi-experimental | 14  5  3 | *63,6*  *22,7*  *13,6* |
| **Tenure^a^**  Renters (not further specified)  Private renters  Public renters | 15  6  2 | *68,2*  *27,3*  *9,1* |
| **Housing insecurity dimension**  Housing instability  Housing affordability  Housing affordability and instability | 13  8  1 | *59,1*  *36,4*  *4,5* |
| **Housing affordability operationalization^a^**  Rent/income ratio (e.g., housing cost burden)  Amount of ‘out-of-pocket rent’/ residual income  Amount of housing benefits  Receiving housing assistance  Living in social housing | 6  3  1  1  1 | *27,3*  *13,6*  *4,5*  *4,5*  *4,5* |
| **Housing instability operationalization^a^**  Forced moves due to legal or economic issues  Eviction  Frequency measure of moving  Risk of eviction  Behind on rent  Crowding/ moving in with others to save costs  Combined measure of residential instability Duration of occupancy | 4  3  3  3  2  2  1  1 | *18,2*  *13,6*  *13,6*  *13,6*  *9,1*  *9,1*  *4,5*  *4,5* |
| **Mental health outcome^a^**  Overall mental health, well being, and psychological distress  Depression  Anxiety  Psychosocial functioning  Mental health treatment  Depression and anxiety combined  Post-traumatic stress disorder  Problematic alcohol use  Suicidal ideation and behavior | 12  10  3  3  2  1  1  1  1 | *54,5*  *45,5*  *13,6*  *13,6*  *9,1*  *4,5*  *4,5*  *4,5*  *4,5* |
| **a** Categories are not mutually exclusive; totals do not apply.  *Notes.* US = United States of America; UK = United Kingdom | | |

**4.** Discussion: an important limitation of the paper is the language chosen by the review. They exist articles in other languages that are lost. The article should mention this limitation.

**Reply:** *Thank you for pointing out this important limitation. We have added it to our limitations.*

**Changes in manuscript (p. 35):** “Fifth, this review is limited to publications in English, potentially overlooking valuable studies in other languages. Although we excluded only two studies based on language, given that our keywords were restricted to English, it is possible that the actual number of papers on this topic is higher.”

Other minor comments:

**5.** Methods: please explain the meaning of “data points”. Does it mean cross sectional data?

**Reply:** *Thank you for pointing out this misleading formulation. We meant by it that studies may include analyses of other populations (e.g., homeowners, children) and research questions (e.g., other housing dimensions) if they provided separate results or analysis for renters. In this case, only data relevant to our research focus on renters was extracted for the review. We specified this in the method section.*

**Changes in manuscript (p. 6f.):** „studies“ instead of „data points“

**Changes in manuscript (p. 7):** “Studies may include analyses of other populations (e.g., homeowners, children) and research questions (e.g., other housing dimensions), if they provided separate analysis for our research focus. However, studies needed to specifically distinguish effects for renters, not simply adjust for tenure or include an interaction.”

**6.** Explain the meaning of “PTSD”.

**Reply:** *Thank you for pointing out the missing definition, which we added in the manuscript.*

**Changes in manuscript (p. 11, 21, 25, 29):** “…post-traumatic stress disorder…”

Carme Borrell

**Reply:** *We hope these revisions adequately address your concerns and enhance the clarity and comprehensiveness of our manuscript. We are grateful for your valuable feedback!*

**Reviewer #2:** This is an important topic with a growing literature, and it is natural to have a review piece that synthesizes the increasing evidence for the relationship between housing insecurity and mental health. The authors identify 22 studies examining the relationship between some measure of housing affordability or stability and mental health. There are several strengths of the review. First, the authors consider multiple dimensions of housing insecurity. The authors also make some attempt to assess the evidence quality or describe the study methodology. Although I think the motivation for a review piece on this topic is clear, I have some concerns about the approach used, and am somewhat skeptical the authors have identified an exhaustive list of studies.

**Reply:** *Thank you for stressing the importance of the topic and the relevance of a systematic review on the relationship between housing insecurity and mental health. We are grateful for the thorough review of the manuscript and appreciate your suggestions and concerns. We have changed the manuscript accordingly with a focus on aiming to strengthen the line of arguments, giving more details about our approach, and – where necessary adding clarifications and limitations. Below, we outline the changes we have implemented in response to your comments in detail:*

Main Concerns

**1.** There are a two aspects of inclusion criteria that were not clear until fairly deep into the paper. First, the authors are only looking at adult outcomes. Second, included studies had to specifically distinguish effects for renters, not simply adjust for tenure or include an interaction. This would be good to mention early on.

**Reply:** *Thank you for your feedback regarding the lack of clarity of our inclusion criteria and the importance of explicitly stating our focus on adult outcomes as well as the requirement for studies to distinguish effects for renters early on. We have adapted the manuscript to clarify these aspects earlier in the paper to enhance transparency and understanding for readers. With regard to the fact that we only look at adult outcomes, this target population is additionally mentioned in the abstract (p. 1), objectives (p. 6), and methods (p. 7).*

**Changes in manuscript (p. 1, abstract):** “Studies needed to specifically distinguish effects for renters, not simply adjust for tenure or include an interaction.”

**Changes in manuscript (p. 3 + p. 4, introduction):** “… adult ...”

**Changes in manuscript (p. 7, methods):** “Studies may include analyses of other populations (e.g., homeowners, children) and research questions (e.g., other housing dimensions), if they provided separate analysis for our research focus. However, studies needed to specifically distinguish effects for renters, not simply adjust for tenure or include an interaction.”

**2.** The number of measures of housing insecurity is almost as high as the total number of included studies. Synthesizing the relationship between the constructs and mental health may be quite difficult with only 22 studies, since the exposures are so varied. If the effect varies across different exposures, it could be difficult to identify without more studies. Limiting to only particular exposures is not ideal, because that would cut the number of studies still. One possibility would be to broaden the criteria to include studies that do not distinguish renters. Another would be to examine other health outcomes beyond mental health. Another would be to include studies of children/adolescents. But this issue should be addressed either up front in the paper or in the discussion.

**Reply:** *Thank you for the important suggestions. We have addressed the number of measures and exposures and the possibilities to broaden the criteria, examine additional health outcomes, or going beyond adult samples, by adding the paragraph below to our discussion section. In addition, we refer to the housing literature to highlight the potential benefit of adopting a unified definition of housing instability and corresponding measures in future studies (p. 32).*

**Changes in manuscript (p. 35):** “Specifically, the operationalizations of housing affordability and instability, as well as mental health outcomes, varied significantly across the included studies (see Tables 1 and 4). However, due to the limited number of studies, discerning which housing insecurity factors and/or mental health outcomes are more or less relevant remains challenging. Confining the analysis to particular variables and operationalizations would have significantly reduced the pool of studies available for analysis. A potential avenue for future research could involve broadening the inclusion criteria to encompass studies that do not differentiate tenure or expanding the range of included studies by including physical health outcomes.”

**3.** No timeframe is given for the literature search, although the earliest study considered was published in 2012. Should this be interpreted to mean that this topic was not studied prior to 2012, or was there an inclusion criterion that excluded earlier studies?

**Reply:** *Indeed no restrictions were made regarding publication date. In our evidence synthesis we pointed out this fact: “No restrictions were made regarding publication date or status.” (page 7) and “With the exception of one study, all were published within the last decade, mostly in 2020 or later.” (page 10). This underscores an interesting trend: while earlier studies were not excluded based on publication date, the majority of relevant research on this topic has emerged in the past decade. To further improve transparency and understanding for the readers, we have added the years of publication in our new descriptive table in response to your commentary.*

**Changes in the manuscript (p. 11):**

|  | **N°**  **(*N* = 22)** | **%** |
| --- | --- | --- |
| **Year of Publication**  2009  2010 – 2019  2020 – 2023 | 1  8  13 | *4,5*  *36,4*  *59,1* |

**4.** I was surprised to find that there were only 11 studies on this topic in the US. I think that may have to do with the inclusion criteria (i.e. this is only a small portion of the overall housing and health literature). In the literature search, was there an indication of which inclusion criterion was excluding most studies? The focus on mental health? The requirement that renters be separate? Adults only?

**Reply:** *This is indeed an interesting observation. Our requirement for separate renter analysis was the main exclusion factor, followed by the absence of mental health outcomes or specific housing insecurity exposure. Traditional literature on housing and health in the US (as well as UK and Australia) has typically focused on homeownership or extreme forms of housing instability/homelessness (both of which were excluded in our review). Consequently, it's accurate to note that our review represents only a fraction of the broader housing and health literature in these countries.*

*Nonetheless, we think that our specific focus on renters holds significant value for several reasons. As you pointed out above, there is a growing body of literature on renters and housing insecurity, particularly given the rising prevalence of renters, especially in urban settings. Concurrently, numerous renters face obstacles in securing suitable, stable, and affordable housing due to various factors such as housing policies, market dynamics, and recent socio-economic crises. This renders them particularly vulnerable to housing insecurity.*

*We specify the reasons for exclusion in the appendix, referenced on page 10: “A detailed overview of the reasons for exclusion is provided in S3 Appendix. The most common reason for exclusion was that studies did not specifically analyze the association of housing insecurity and mental health among renters but rather addressed housing insecurity more broadly (e.g., statistical analyses were not separated by tenant subgroups).”*

**5.** The authors assess strength of evidence, but they do not explicitly consider the causal nature of evidence presented in the included studies. It would be useful to have some sense of whether the comparison group used in the study represents a rigorous attempt to assess the causal effects of housing insecurity.

**Reply:**  *Thank you for highlighting the importance of considering causality in more detail in our assessment of the evidence presented in the included studies. In response to your feedback, we have made significant revisions to enhance transparency regarding the methodologies utilized in the included studies, particularly those utilizing longitudinal and quasi-experimental designs where causal inference is pertinent. Specifically, we have added a paragraph to the results section, elaborating on the methodologies utilized, such as regression models with within-person effects, marginal structural models, matching control groups, and difference-in-difference designs, which enhance causal inference. Additionally, we have incorporated another paragraph into the discussion section, discussing the strengths and weaknesses of the included studies in relation to causality. We believe that this addition will contribute to a clearer understanding of the strength of evidence regarding the possibility to draw causal effects of housing insecurity. Thank you for your valuable feedback.*

**Changes in the manuscript (p. 26f.):** “The studies employed various statistical models based on their design and dataset, with many utilizing robust methods to adjust for confounders such as multivariable regression, matching, and propensity score matching. For instance, most longitudinal studies used some form of regression analysis with fixed effect, enabling within-person comparisons over time and controlling for time-invariant individual characteristics (Bentley et al., 2016; Elliott et al., 2021; Leifheit et al., 2021; A. Li et al., 2022; Mason et al., 2013; Park & Seo, 2020; Pevalin, 2009; Rodgers et al., 2019; ViforJ et al., 2022). A. Li et al. (2022) employed a marginal structural model to address time-varying confounding variables and selection biases by weighting each observation, ensuring comparability between exposed and unexposed groups. Several studies combined regression analysis with matching methods, selecting the sample so to achieve covariate balance between exposed and unexposed groups (Desmond & Kimbro, 2015; H. Kim & Burgard, 2022; Prentice & Scutella, 2020; Reeves et al., 2016). Reeves et al. (2016) used a regression model that included a difference-in-difference design and Ali and Wehby (2022) a triple-difference design, offering control over potentially unobserved confounding factors by comparing treatment and control groups before and after an intervention or treatment. All cross-sectional studies used some form of regression analysis and controlled for multiple covariates (Acharya et al., 2022; Burgard et al., 2012; Chambers et al., 2015; Sandel et al., 2018; C. Vásquez-Vera et al., 2022), with three studies omitting income as an important potential confounder (Chambers et al., 2015; Sandel et al., 2018; C. Vásquez-Vera et al., 2022). Notably, two longitudinal studies solely conducted bivariate analyses without accounting for potential confounders, which indicates a significant risk of bias (Baker, Lester, et al., 2020; J. Tsai et al., 2021). Arundel et al. (2022) did not specify the statistical methods utilized.”

**Changes in the manuscript (p. 32):** “…and quasi-experimental designs (*n* = 3).”

**Changes in the manuscript (p. 33):** “…The included cross-sectional studies, in particular, are susceptible to biases due to the lack of temporality, confounding, selection bias, and endogeneity, which hinder causal interpretation. However, this review incorporates also numerous studies utilizing robust methodologies like longitudinal studies with regression analysis with fixed effects, marginal structural models, or quasi-experimental designs for both dimensions of housing insecurity. In the absence of randomized controlled trials, these approaches improve causal inference by addressing time-varying and invariant confounders and establishing temporal precedence. Nevertheless, the possibility of unobserved confounders and reverse causation persists.”

**6.** Is it useful to examine studies across so many countries, especially when the included countries have so few studies? It would be one thing if the search identified 10 studies from every country. But it is not clear that having one study from South Korea, one from Spain etc. is informative, particularly if the relationship between housing and health differs at all across countries. Since nearly all studies were from the US, UK, and Australia, the review could be limited to the anglosphere without losing much of the sample. At the very least, there should be a strong rationale for the inclusion of studies from all OECD countries.

**Reply:** *Thank you for your thoughtful considerations regarding the inclusion of all OECD countries in our systematic review. When we conducted the search, we applied broad inclusion criteria because the distribution of studies across countries was not known to us (although of course, we expected a higher number of studies from countries like the UK, US, and Australia due to their robust literature on housing and health). After executing the search, we chose not to alter our criteria for several reasons:*

- *Including all OECD countries provides valuable insights into the state of research globally in countries that at the same time share some economic, social, and policy characteristics, highlighting both areas of strength and gaps in knowledge. The predominance of studies from anglophone countries underscores a significant research gap, emphasizing the need for more diverse geographical representation in future studies (see page 33f.).*
- *Despite the limited number of studies from non-anglophone countries, their findings align with trends observed in anglophone countries, suggesting a comparable relationship between rental insecurity and mental health outcomes (see page 33f.).*
- *While specific policy contexts and societal factors may vary across OECD countries (e.g., welfare state models and tenure structures), they share common economic, social, and policy characteristics that can influence the relationship between housing insecurity and mental health.* *For instance, OECD countries typically exhibit similar economic structures characterized by market-based economies with varying levels of government intervention, and they often adopt comparable housing policies and regulations, albeit with variations in their scope and implementation (e.g., social housing programs, tenant protections). Additionally, overarching trends such as the impact of minimal government intervention in housing markets are evident across OECD countries in the last decades. Consequently,* *the results of the review hold relevance for all OECD countries and to some extent, beyond them.*

*Nevertheless, generalizability is impeded, and studies need to be analyzed at an individual level. This is one reason why we performed a narrative synthesis instead of a meta-analysis. To better explain the decision to include the OECD countries, we have added the following paragraph to our introduction in response to your feedback:*

**Changes in manuscript (p. 3):** “Housing insecurity is profoundly influenced by various macrostructural factors, including the welfare state, housing system, public housing policies, and market dynamics (Marí-Dell'Olmo et al., 2017). Despite the diverse housing systems, regulatory environments, and socio-economic factors among countries, many advanced economies share a common trajectory of severe housing insecurity challenges (Lee et al., 2022). This review focuses on OECD countries for several reasons: They typically exhibit similar economic structures characterized by market-based economies with varying levels of government intervention, and they often adopt comparable housing policies and regulations, albeit with variations in their scope and implementation, such as social housing programs and tenant protections. Furthermore, in many OECD countries, heavy reliance on the market for access to housing has led to rising prices and reduced accessibility of housing, exacerbating the challenges for renters in securing affordable, stable, and suitable housing (Baumgartner et al., 2023; Bone, 2014; Clair et al., 2019; Eurostat, 2022; Routhier, 2019) (…) Moreover, public expenditure on housing remains low across many OECD countries, resulting in a heavy reliance on the often minimally regulated private rental market for access to affordable housing (Del Salvi Peroi et al., 2016). Although some governments have implemented a range of housing policies and programs to address the issue of housing security, including social/public housing, housing subsidies, or eviction policies, these initiatives frequently fail to reach those who truly need them, leaving many eligible renters without the necessary support (DeLuca & Rosen, 2022; Dweik & Woodhall-Melnik, 2022) (…) within OECD countries. “

*We hope we have addressed your concerns satisfactorily. Thank you again for your valuable feedback! Your contributions have helped us clarify important aspects and strengthen the overall presentation of our research.*
