# Supplementary material for: Exploring the association between housing insecurity and mental health among renters: A systematic review of quantitative primary and secondary studies
Source: PLoS One. 2026 Jan 16;21(1):e0294222. doi: 10.1371/journal.pone.0294222 (PMC12810827; doi:10.1371/journal.pone.0294222)
Supplement: Attachment — Submitted filename: Reply letter_R3.docx [file pone.0294222.s010.docx]

**PONE-D-23-35202_R3**

**Exploring the association between housing insecurity and mental health among renters: A systematic review**

Dear Dr. Clemence,

Dear Dr. Carswell,

Dear Reviewer,

Thank you for your feedback on our manuscript and the additional communication. We would like to reply to the reviewer and to the additional communication in this rebuttal letter.

We still think that the whole review process has not been transparent and comprehensible for us as authors. Probably, we would have preferred to receive the expert’s opinion during the first additional revision after the formal acceptance of the manuscript. However, since we have already put a lot of efforts into the manuscript and think that it targets an important topic, we would like to submit a revised version of the manuscript. As suggested, we decided not to update the literature search since we think that we submitted the review timely after the search.

I am very sorry that the submission of the revised manuscript has been delayed on my part. A long period of sick leave did not allow me to complete the revision earlier.

Now, we would like to respond to the reviewer’s comments:

PLOS ONE

[Note: HTML markup is below. Please do not edit.]

Reviewers' comments:

Reviewer's Responses to Questions

**Comments to the Author**

1. If the authors have adequately addressed your comments raised in a previous round of review and you feel that this manuscript is now acceptable for publication, you may indicate that here to bypass the “Comments to the Author” section, enter your conflict of interest statement in the “Confidential to Editor” section, and submit your "Accept" recommendation.

Reviewer #3: (No Response)

2. Is the manuscript technically sound, and do the data support the conclusions? The manuscript must describe a technically sound piece of scientific research with data that supports the conclusions. Experiments must have been conducted rigorously, with appropriate controls, replication, and sample sizes. The conclusions must be drawn appropriately based on the data presented.

Reviewer #3: Partly

3. Has the statistical analysis been performed appropriately and rigorously?

Reviewer #3: N/A

4. Have the authors made all data underlying the findings in their manuscript fully available?

The PLOS Data policy requires authors to make all data underlying the findings described in their manuscript fully available without restriction, with rare exception (please refer to the Data Availability Statement in the manuscript PDF file). The data should be provided as part of the manuscript or its supporting information, or deposited to a public repository. For example, in addition to summary statistics, the data points behind means, medians and variance measures should be available. If there are restrictions on publicly sharing data—e.g. participant privacy or use of data from a third party—those

must be specified.

Reviewer #3: Yes

5. Is the manuscript presented in an intelligible fashion and written in standard English? PLOS ONE does not copyedit accepted manuscripts, so the language in submitted articles must be clear, correct, and

unambiguous. Any typographical or grammatical errors should be corrected at revision, so please note any specific errors here.

Reviewer #3: Yes

6. Review Comments to the Author

Please use the space provided to explain your answers to the questions above. You may also include additional comments for the author, including concerns about dual publication, research ethics, or publication ethics. (Please upload your review as an attachment if it exceeds 20,000 characters)

Reviewer #3: GENERAL COMMENTS

Thank you for the opportunity to review this revised systematic review titled “Exploring the association between housing insecurity and mental health among renters: A systematic review”. I note here that I have not reviewed this manuscript before and did not look at the prior reviewer’s comments so as to not bias my own review. While I think this is a critically important topic and is generally well done, I have several suggestions that I believe will strengthen this work, detailed in my specific comments that follow. The two major issues that I see are the need to update the search for potentially eligible studies since it is almost two years old now, as well as the need to use a formal instrument to assess the strength/ certainty of evidence. I focus my review primarily, but not exclusively, on the systematic review methodology, something that inextricably affects the results and discussion sections of any type of study.

I am hopeful that my comments will be taken in the spirit of improving this work, with the specific goal of advancing the field. Also, please note that my comments are focused on the clean copy of the revised manuscript.

Thank you very much for taking the time to review our manuscript. The constructive suggestions have helped us to improve the manuscript considerably. And yes, we took all of your feedback as a chance to improve our work and to contribute to the field of study. Further, we appreciate your concerns to review only the clean version of the manuscript and not read the previous reviews. However, we think it would be helpful to give some context to the review process so far. Our manuscript had been formally accepted in July 2024 and the Editor decided afterwards to retract the acceptance. Since then, we have put a lot of efforts into the improvement of the manuscript. This is now the third round of revision, each with different requirements and partly contradicting suggestions to previous reviews. With this history of revision process in mind, we will answer to your suggestions.

SPECIFIC COMMENTS

*Title page – Per PRISMA 2020 guidelines, please include the type(s) of study design(s) that were eligible in your title.

Thank you for your feedback, we changed the title accordingly:

*Exploring the association between housing insecurity and mental health among renters: A systematic review of quantitative primary and secondary studies* (Titlepage, l. 8-10)

*Page 1, lines 24 through 49 (Abstract) – First, while working within the guidelines of the journal, please revise your abstract in accordance with PRISMA 2020 guidelines, void of the meta-analytic piece (See, for example: Page MJ, Moher D, Bossuyt PM, et al. PRISMA 2020 explanation and elaboration: updated guidance and exemplars for reporting systematic reviews. BMJ. 2021;372:n160. https://www.bmj.com/content/bmj/372/bmj.n160.full.pdf).The 12-item checklist (Box 2) on the third page of the PRISMA guidelines should be helpful to you.

Thank you very much for your detailed feedback and guidance. We changed the abstract accordingly and indicated which information refers to which of the 12 items of the checklist (p.1 – 2, l. 24 – 62).

Second, on line 25, suggest that you delete the word “first” or say something like “To the best of the authors knowledge, this is the first…” My main point here is that using the term “first” is usually too verbose given the margin of search error when trying to identify related systematic reviews on a topic.

Thank you for this important hint. We changed the sentence as suggested:

“*To the best of the authors’ knowledge, the present systematic review is the first to investigate the association between housing insecurity and mental health outcomes among renters, with a focus on housing affordability and instability.”* (p. 1, l. 25 – 28)

Third, on lines 28 and 29 I see that the searches were conducted in December of 2022. Since the search for studies is approximately two years old, and despite the fact that you performed forward citation tracking up to February 27, 2023, the search for potentially eligible studies probably needs to be updated.

Thank you for the feedback on the date of our searches. We agree that the searches could benefit from an update. Due to the long review process and the different obstacles, we have been facing during the process, we decided to accept the Editor’s offer not to update the searches.

Fourth, and per PRISMA guidelines, you should briefly report the results of your risk of bias assessments. You should probably also assess the certainty of evidence using something like the Grading of Recommendations Assessment, Development, and Evaluation (GRADE) instrument. Guidelines for doing so when a meta-analysis is not conducted are available here: Murad MH, Mustafa RA, Schünemann HJ, Sultan S, Santesso N. Rating the certainty in evidence in the absence of a single estimate of effect. Evidence Based Medicine. 2017;22(3):85-87. https://ebm.bmj.com/content/ebmed/22/3/85.full.pdf.

Thank you for your recommendation. We applied the GRADE instrument to assess certainty of evidence for each mental health outcome according to the procedure recommended by Murad et al. (2017). All outcomes were rated independently by two raters. We describe the procedure in the method section (p. 11, l. 293 – 306) and present the results in the results section (p. 31 – 34, l. 471 – 527).

Further, we added the respective information to the Abstract:

“*The methodological quality of the included studies was rated with the JBI Critical Appraisal Tools, and the certainty of evidence was rated using the Grading of Recommendations Assessment, Development, and Evaluation (GRADE) framework.*” (p. 2, l. 38 – 41)

“*Based on the JBI ratings, the overall methodological quality of the included studies was good. The overall ratings of certainty of evidence, based on the GRADE ratings, were between low and very low – mainly due to the non-controlled study designs of included studies*.” (p. 2, l. 45 – 47)

Finally, your conclusions should be tempered by the certainty of evidence and most likely the need for future well-designed studies on this topic.

Thank you for this important suggestion. We used the results of the GRADE rating to discuss the findings and our conclusions accordingly (compare p. 37 – 47). Based on a mostly low certainty of evidence, we now emphasize the need for future well-designed studies. We revised the abstract accordingly:

*“Despite methodological limitations due to the non-controlled studies included in the review, the findings suggest overall that experiencing unaffordable or unstable housing has a negative impact on renters’ overall mental health and depressive symptoms. Housing insecurity poses a significant challenge for renters in OECD countries, highlighting the need for policymakers to implement supportive housing policies and tenure protection measures in order to improve renters’ housing security and ultimately public health. Nevertheless, more research with robust study designs is needed to draw further conclusions.”* (p. 2, l. 54 – 61)

*Pages 2 through 5, lines 51 through 150 (Introduction) – Overall, a nice Introduction section.

Thank you for your positive feedback on our Introduction section.

*Page 6, lines 163 through 167 (Methods) – Please tell the reader if the decision to conduct a systematic review without a meta-analysis was made a priori or post hoc. Along those lines, throughout the rest of your Methods section, please tell the reader what post hoc changes were made to your a priori protocol as well as the reasons for such. This is of course assuming that you had an a priori protocol.

Although we did not publish a study protocol or a preregistration, we followed a strict a priori defined procedure during the systematic review. It was determined a priori that the decision on whether to conduct a meta-analysis would be made post hoc after reviewing the suitability of the data:

“*Based on our knowledge of the field of research and anticipated heterogeneity in study methodologies and definitions of housing insecurity, we decided a priori that a meta-analysis would be considered if sufficient homogeneity was found—which was ultimately not the case. Therefore, a narrative synthesis was chosen over a meta-analysis to synthesize the evidence.*” (p. 12, l. 311 – 314)

*Pages 6 and 7, lines 169 through 193 (Methods, Eligibility criteria) – First, please make it clearer to the reader if eligible studies were limited to those published in peer-reviewed journals or studies from other sources (masters theses, dissertations, abstracts from conference proceedings, etc.) were also eligible.

Thank you for pointing this out. We added more specific information regarding the publication status of eligible studies to the manuscript:

“*No restrictions were made regarding publication date or status including published and unpublished studies, partially published studies, and studies from other sources like masters’ theses, dissertations, abstracts from conference proceedings, not peer reviewed.”* (p. 8, l. 204 – 207)

Second, in the first paragraph, you use the term “such as” in multiple places. However, your eligibility criteria should be more definitive.

Thank you for this suggestion. We aimed at giving examples to illustrate the respective inclusion criteria. We changed the wording accordingly and added the relevant operationalization of the respective construct in parenthesis:

“*To be eligible for inclusion, studies needed to be quantitative primary or secondary research that investigated housing insecurity by examining at least one variable related to housing affordability (housing cost burden, housing affordability stress, receiving rental assistance, or living in social housing), and/or housing instability (frequent moves, forced moves, risk of eviction, behind on rent, or overcrowding). Studies investigating housing programs or policies aimed at improving affordability and/or stability (rental assistance or eviction prevention programs), were also considered for inclusion. Furthermore, studies needed to include at least one mental health-related outcome (specific mental disorders, symptoms of psychological distress, overall mental health and well-being, quality of life, psychosocial functioning, suicidal behavior, or treatment for mental health reasons, e.g., medication, outpatient, or inpatient therapy for emotional conditions).*” (p. 7 – 8, l. 190 – 200)

*Pages 7 and 8, lines 195 through 216 (Methods, Search Strategy) – Please tell the reader who was responsible for conducting the database searches. Also, and as previously mentioned, the search for studies should be updated.

Thank you for your recommendations. As described above, we decided not to update the search for studies. However, we stated explicitly the responsibility for conducting the database searches:

“*A comprehensive search of the literature was conducted in two stages by the first author M.T. under the supervision of the last author H.N.*” (p. 8, l. 220 – 221)

*Page 8, lines 218 through 231 (Methods, Study Selection) – First, on line 219, please tell the reader if duplicates were removed electronically and/or manually.

Thank you for this recommendation. We added the information as suggested:

“*After exporting all search results into the reference management program Citavi (Version 6.14), duplicate records were removed manually by the first author M.T.*” (p. 9, l. 244 – 245)

*“The initial search yielded a total of 1,198 potentially eligible titles (**after the first author M.T. had removed duplicates manually).”* (p. 12, l. 321 – 323)

Second, on lines 230-231, the use of Gwet’s AC1 statistic may be preferable to Cohen’s kappa. See, for example: 1. Gwet KL. Computing inter-rater reliability and its variance in the presence of high agreement. Br J Math Stat Psychol. 2008;61(Pt 1):29-48.

Thank you for this helpful suggestion. We followed your advice and calculated Gwet’s AC1 instead of Cohen’s Kappa:

*“Additionally, to ensure reliability, a randomized subset of 10 % of the full texts (n = 12) was independently screened by the author L.N. and the interrater reliability was calculated using Gwet’s AC_1_ [58] and the R package irrCAC [59].”* (p. 10, l. 255 – 258)

*“The interrater reliability of the study selection process was Gwet’s AC1 = .906, indicating high agreement [58,59].”* (p. 13, l. 332 – 333)

*Pages 8 and 9, lines 233 through 255 (Methods, Data Extraction and Synthesis Method) - Please tell the reader if any attempt was made to retrieve missing data from investigator. If not, then this should also be stated. If missing data were requested, the process for doing so should be described.

Thank you for suggesting to add this important information. We added it to the “Data Extraction and Synthesis Method” section:

“*In cases where data or information was missing or unclear, study authors were contacted several times by email to obtain or clarify missing data or unclear information. It was marked as not available (N/A) if it could not be obtained by contacting the study authors.”* (p. 10, l. 269 – 272)

Also, on line 246, interrater agreement may also be better assessed using Gwet’s AC1 statistic.

Thank you for this advice. We changed the procedure and manuscript accordingly:

*“The interrater agreement was measured using Gwet’s AC1 (58) and the R package irrCAC (59).”* (p. 11, l. 276 – 277)

*“The interrater agreement for data extraction was Gwet’s AC1 = .914, indicating high agreement.”* (p. 13, l. 336 – 338)

*Pages 9 and 10, lines 257 through 263 (Methods, Reporting Quality) – First, suggest that you retitle this subsection as “Risk of bias assessment”. Second, why did you not use something like the MASTER scale, an assessment instrument that allows for the evaluation of all the different types of study designs you included versus two separate instruments? See: 1. Stone JC, Glass K, Clark J, et al. The MethodologicAl STandards for Epidemiological Research (MASTER) scale demonstrated

a unified framework for bias assessment. J Clin Epidemiol. 2021;134:52-64. Doi: 10.1016/j.jclinepi.2021.01.012; 2.

Ahmed AI, Kaleem MZ, Elshoeibi AM, et al. MASTER scale for methodological quality assessment: Reliability assessment and update. 2024. J Evid Based Med. doi: 10.1111/jebm.12618.

Thank you for your feedback regarding the assessment of the methodological quality and risk of bias assessment. In the initially submitted version of the manuscript, we assessed the reporting quality (which is why the subsection had the respective title). After the first revision and formal acceptance of the manuscript, the Editor asked us to rate the methodological quality of included studies instead of the reporting quality. We therefore searched for useful and established instruments that also suit the characteristics of our review and the studies included. We used the repository provided by the Editor (<https://osf.io/dmrq6>) and the following literature (Ma et al., 2020; Mamikutty et al., 2021) to make our decision, among others. Based on this information and our joint experiences with systematic reviews and meta-analyses, we decided to use the JBI appraisal tools. Before applying the instrument, we communicated with the Editor and asked for her opinion on the JBI appraisal tools and their usefulness. She agreed and informed us that the journal will not give us instructions which tool we should use. She informed us that “*PLOS ONE does not require that authors use any specific quality assessment tools and we would have no concerns about the use of the JBI appraisal tools, provided they are used correctly.*” – which is why we expected that we will only receive feedback on the quality of application not the instrument per se. As we assessed the quality of the studies and not the risk of bias, we will refrain from retitling the section.

The MASTER scale is not mentioned in any of these sources mentioned above, which is why we did not consider the scale at all. We think it might be confusing for the reader to add information regarding the MASTER tool to our manuscript since we did not use it. We hope that you can understand our decision and the process that lead to it.

Ma, L. L., Wang, Y. Y., Yang, Z. H., Huang, D., Weng, H., & Zeng, X. T. (2020). Methodological quality (risk of bias) assessment tools for primary and secondary medical studies: what are they and which is better? *Military Medical Research, 7*, 1-11. <https://doi.org/10.1186/s40779-020-00238-8>

Mamikutty, R., Aly, A. S., & Marhazlinda, J. (2021). Selecting risk of bias tools for observational studies for a systematic review of anthropometric measurements and dental caries among children. *International journal of environmental research and public health, 18*(16), 8623.

Third, on line 263 interrater agreement may also be better assessed using Gwet’s AC1 statistic.

Again, we decided to follow your advice and calculated Gwet’s AC1:

*“Disagreement was resolved through discussions between the authors and the interrater agreement was measured using Gwet’s AC1 (58) and the R package irrCAC (59)”* (p. 11, l. 293 – 295)

*“Interrater agreement for the critical appraisal of the methodological quality was Gwet’s AC1 = .872, indicating high agreement.”* (p. 29, l. 399 – 400)

After line 263, two other titled subsections should probably be added. The first should be titled something like “Strength/ Certainty of Evidence” and assessed using something like the previously mentioned Grading of Recommendations Assessment, Development, and Evaluation (GRADE) instrument. Guidelines for doing so when a meta-analysis is not conducted are available here: Murad MH, Mustafa RA, Schünemann HJ, Sultan S, Santesso N. Rating the certainty in evidence in the absence of a single estimate of effect. Evidence Based Medicine. 2017;22(3):85-87. https://ebm.bmj.com/

content/ebmed/22/3/85.full.pdf. Please note that this section is different than the assessment for risk of bias. In fact, risk of bias assessments feed into the GRADE assessment. Also, more than one person should be responsible for assessing the strength/certainty of evidence.

Thank you very much for the feedback and detailed instruction. We applied the GRADE rating as suggested with two independent raters. We partly used the information of the JBI rating for the GRADE rating. We describe the procedure in the method section (p. 11, l. 293 – 306) and present the results in the results section (p. 31 – 34, l. 471 – 527). The detailed rating can be found in S6 Appendix of the manuscript.

*“Certainty of Evidence*

*To assess the certainty of evidence regarding the effects of housing affordability and housing instability on different mental health outcomes, we applied the Grading of Recommendations Assessment, Development, and Evaluation (GRADE) instrument for observational studies [61]. First of all, we assessed the number of studies, the overall sample size, and the qualitatively summarized effect of housing affordability or housing instability on the respective mental health outcome. The risk of bias assessment was mainly based on the JBI quality ratings. Afterwards, we rated the inconsistency, indirectness, imprecision, possible publication bias, and factors that can raise the certainty in evidence. According to the GRADE guidance, certainty of evidence of observational, non-controlled trials is always rated as low certainty, and can be upgraded or downgraded based on the individual ratings on the subscales [62]. GRADE ratings were independently assessed by the authors L.N. and M.T. Disagreements were discussed until agreement was reached. The interrater agreement was measured using Gwet’s AC1 [58] and the R package irrCAC [59].”*

*“Certainty of evidence*

*A detailed evaluation of every item of the GRADE rating for each mental health outcome is provided in S6 Appendix. Interrater agreement was Gwet’s AC1 > .999, indicating high agreement. In total, the certainty of evidence for the 12 mental health outcomes was rated as low or very low, respectively. This overall low to very low certainty of evidence is largely a result of the study designs rather than poor quality per se. In the GRADE framework, non-randomized, observational studies are initially rated as low certainty and can only be upgraded under specific circumstances [62].*

*Studies on housing affordability investigated overall mental health (five studies), depression (four studies), and psychosocial functioning (one study). Overall mental health and depression were rated as low certainty. Despite small numbers of studies for these outcomes, sample sizes of both outcomes were high, and populations and measures were diverse – which seems reasonable in the context of housing insecurity. Still, the majority of studies pointed in the same direction with similar effects. Only one study investigated the effect of housing affordability on psychosocial functioning. Although the study is of high methodological quality, the sample size is small and the population under study very specific. Therefore, we rated the overall certainty of evidence as very low.*

*Studies on housing instability investigated overall mental health (seven studies), depression (seven studies), anxiety (three studies), psychosocial functioning (two studies), mental health treatment use (two study), depression and anxiety combined (one study), posttraumatic stress disorder (one study), problematic alcohol use (one study), and suicidal ideation (one study). We rated the certainty of evidence regarding overall mental health as low. Only two studies were of high methodological quality, but the overall sample size was large, and effects were generally consistent across populations and operationalizations of housing instability. Effects mostly point in the same direction of a small negative effect of housing instability on overall mental health with little variability (i.e., small CIs). For depressive symptoms in the context of housing instability, we also rated the certainty of evidence as low. Six out of seven studies show high methodological quality and the other aspects of the rating show no or borderline concerns. Most of the studies reported small negative effects of housing instability on depressive symptoms. Still, since no factors contribute to formally upgrade the level of certainty of evidence, it remains a low certainty rating. The three studies addressing housing instability and anxiety show different study designs and mixed results. We rated the certainty of evidence as low. All three studies are of high methodological quality. At the same time, they applied different methodological approaches in different populations, making a direct comparison and aggregation of their findings difficult. No factors contributed to an upgrading or downgrading of the certainty rating. We rated the certainty of evidence of the included studies investigating housing instability and psychosocial functioning as low. Only two studies investigated psychosocial functioning as mental health outcome but with a large total sample size. The populations under study and methods applied are heterogeneous. Nevertheless, for two out of three outcome measures a small negative effect of housing instability on psychosocial functioning was reported. The studies controlled adequately for confounding factors, but only one study reported (moderate) confidence intervals. Two studies addressed the relation between housing instability and mental health treatment use with an overall very low certainty of evidence. Only one study showed a high methodological quality. We assessed (very) serious concerns regarding study quality, inconsistency, and borderline concerns regarding imprecision which led to the downgrading of the certainty of evidence of the outcome mental health treatment use. All other outcome measures in the context of housing instability (depression and anxiety combined, posttraumatic stress disorder, problematic alcohol use, and suicidal ideation) have been studied by only one study each and no significant effect has been reported. Depression and anxiety combined, posttraumatic stress disorder, and suicidal ideation have been part of the same longitudinal study with small sample size (N = 89) and moderate methodological quality. Therefore, the evidence of the three outcomes shows the same methodological limitations which led to the same rating of very low certainty of evidence in all three cases. Problematic alcohol use has been studied by one cross-sectional study. We rated (very) serious concerns regarding study quality, imprecision, and publication bias leading to an overall rating of very low certainty of evidence regarding housing instability and problematic alcohol use (for details see Appendix S6).”*

The second subsection that should be added could be titled something like “Research Synthesis”. This is where you describe the fact that you analyzed your results qualitatively, i.e., without a meta-analysis, as well as telling the reader

why you did not conduct a meta-analysis. You should also tell the reader that you limited your statistics to those that were descriptive in nature, i .e., frequencies and percentages. Some of this information was previously described earlier in your Methods but should be moved to this final subsection of your Methods.

Thank you for your suggestion. We moved the information regarding our analysis to the new section “Research Synthesis” and added more details about the narrative synthesis:

*“Research Synthesis*

*Due to the heterogeneity of the included studies regarding methodology and the applied definitions of housing insecurity, a narrative synthesis was chosen over a meta-analysis to synthesize the evidence. This decision was made post hoc after study selection was completed based on the included studies. In the results section, we will describe the characteristics and methods applied by the included studies, and their methodological quality. Further, we will report descriptive (e.g., frequencies, percentages) and inferential statistics (e.g., results from correlational analyses, regression analyses), and summarize them qualitatively.”* (p. 12, l. 310 - 318)

*Page 10, line 264 – Strongly suggest that you replace the title “Evidence Synthesis” with “Results”.

Thank you for this suggestion. We changed the headline accordingly (p. 12, l. 319).

*Page 10, line 276 (Study Selection) – Here and throughout the rest of the manuscript, please reference the 22 studies that were included, whether discussed in part or in full.

Thank you for your feedback. We added the specific references throughout the whole manuscript.

*Table 1, pages 11 and 12 – This is a nice table.

Thank you for the positive feedback on the table.

*Table 2, pages 13 through 16 – This is a nice table. However, I would suggest that you also add a column that includes any funding that these studies received.

Thank you for this suggestion. We added a respective column to Table 2 and Table 3.

*Table 3, pages 17 through 23 – This is another nice table. My only suggestion is that for each study you delineate exactly what variables the original study authors considered to be primary as well as what variables were considered secondary.

Thank you for this suggestion. We checked all 22 studies again for this information. Actually, most of the studies only reported the independent and outcome measures also reported in Table 2 and 3. For all studies that explicitly defined variables as primary or secondary, we added the respective information to Table 2 and 3. Many included studies are secondary analyses based on larger surveys, and selected the variables of interest and some covariates from these surveys without further specifying.

*Table 4, pages 24 and 25 – This is yet another nice table.

Thank you again for the positive feedback.

*Pages 24 through 31, lines 309 through 477 (Results) – With the exception of the need for better referencing, this section of results are well-described

Thank you for your feedback on the content of our results section. We added the references as suggested.

*Pages 31 through 37, lines 479 through 640 (Discussion) – For ease of reading, suggest that you partition your Discussion into the following subsections and discuss: (1) Overall findings, (2) Implications for Research, (3) Implications for Practice, (4) Implications for Policy (if any), and (5) Strengths and Potential Limitations. Also, your discussion regarding the strength of evidence on pages 33 through 34 (lines 524 through 567) would be strengthened by formal assessment using something like the GRADE instrument I recommended earlier in my review.

We appreciate your suggestions regarding the headings and order of subsections in the discussion section of our manuscript. We changed the headings accordingly and changed the order, first presenting overall findings and implications and afterwards the strengths and limitations of the evidence and of our study.

We also applied the GRADE rating as recommended and discussed its results accordingly.

“*Our conclusions are mainly based on studies with overall good methodological quality (rated with the JBI appraisal tools), but overall low certainty of evidence (rated with GRADE). Conclusions must therefore be interpreted with caution. However, the low GRADE rating is partly a result of the conservative nature of the GRADE framework, which classifies observational studies—common and often necessary in this field—as low certainty by default. While this conservative approach supports robust conclusions in clinical research based on randomized controlled trials ([compare 92)], it may undervalue the strength of evidence in contexts like housing instability, where experimental designs are rarely feasible. Many of the included studies employed longitudinal or prospective designs, or were based on large, nationally representative data sets, which supports their findings despite some methodological limitations. Further, methodological quality of many included studies was rated as overall good when using JBI critical appraisal tools for the respective study design. In general, many studies on housing unaffordability—both included and not included in the current review—point in the same direction. Nonetheless, to more firmly establish this relationship and particularly to assess the impact of targeted interventions, future studies with rigorous and transparent methodological approaches are needed. While current findings offer important insights, conclusions should remain tentative until further high-quality evidence is available.*” (p. 38, l. 643 – 659)

“*Again, the evidence examining housing instability and mental health outcomes were both rated with overall good methodological quality (based on JBI ratings) and (very) low certainty, especially for the outcomes based on only one study (based on GRADE ratings). This partly limits the validity of our conclusions. Nevertheless, the downgrading of the certainty of evidence was mainly based on the non-randomized, non-controlled study designs of included studies. Still, several studies show moderate to high methodological quality and some studies with large sample sizes point into the same direction of a negative effect. Certainty of evidence for housing instability and depression seems to be the most robust evidence in the current systematic review. Six out of seven studies show high methodological quality and the other aspects of the rating show no or borderline concerns. Most of the studies reported small negative effects of housing instability on depressive symptoms. Still, since no factors contribute to formally upgrade the level of certainty of evidence, it remains a low certainty rating. Thus, although findings and conclusions need to be interpreted against the background of general low certainty of evidence, a negative effect of housing instability and mental health is more likely than no effect.”* (p. 39 – 40, l. 674 – 688)

“These conclusions are mainly based on evidence with acceptable and good methodological quality (based on JBI ratings), whose certainty of evidence has been rated as (very) low (using GRADE ratings). Nevertheless, single large, longitudinal studies with robust methodology point into the same direction of negative associations between housing insecurity and mental health. Further, our understanding of individual factors contributing to mental health problems is much more advanced than our knowledge regarding structural determinants of mental health and from a less individualized point of view. Thus, more research and development of robust study designs and statistical approaches are necessary, to improve certainty of evidence in this field of research.” (p. 42, l. 742 – 750)

*Page 37, lines 642 through 650 (Conclusions) – First, and as previously mentioned, I would delete the word “first” on line 642 or revise to say something like “To the best of the authors knowledge, this is the first systematic review…”

Thank you very much. We changed the wording in both the Abstract and in the Conclusions.

Second, and as also previously mentioned, I think your Conclusions need to be tempered after formal assessment of the strength/ certainty of evidence and most likely the need for future well-designed studies on this topic.

Thank you very much for this suggestion. As described above, we applied the GRADE rating as suggested. We also adapted the discussion and conclusions accordingly while still recognizing the strengths of the evidence (compare p. 37 – 47).

*Pages 38 through 44 (References) – Suggest that you place your references in the same font and size as your manuscript, adhere to the journal guidelines, and be consistent. For example, some journal titles are spelled out in full while others are abbreviated.

Thank you very much for this comment. We checked the References and harmonized the formatting according to PLOS ONE’s guidelines.

*S1 (PRISMA Checklist) – Some items in the manuscript and other materials are either not reported or not reported in sufficient detail.

Thank you for this comment. We tried to add more information to the PRISMA checklist and be more precise, especially when we have previously stated “N/A”. We also incorporated the additions and clarifications prompted by your comments above. We hope these amendments address the concerns raised; however, please let us know if any critical aspects remain unclear

END OF REVIEW

Thank you very much for your detailed feedback on the manuscript. We are convinced that the manuscript has improved considerably based on your comments.

Sincerely yours,

Mira Talmatzky & Laura Nohr

On behalf of all Co-Authors
